# Multi-Scale Analysis of Knee Joint Acoustic Signals for Cartilage Degeneration Assessment

**DOI:** 10.3390/s25030706

**Published:** 2025-01-24

**Authors:** Anna Machrowska, Robert Karpiński, Marcin Maciejewski, Józef Jonak, Przemysław Krakowski, Arkadiusz Syta

**Affiliations:** 1 Department of Machine Design and Mechatronics, Faculty of Mechanical Engineering, Lublin University of Technology, Nadbystrzycka 36, 20-618 Lublin, Poland; 2 Department of Electronics and Information Technology, Faculty of Electrical Engineering and Computer Science, Lublin University of Technology, Nadbystrzycka 36, 20-618 Lublin, Poland; 3 Department of Trauma Surgery and Emergency Medicine, Medical University of Lublin, Staszica 11, 20-081 Lublin, Poland; 4 Orthopaedic and Sports Traumatology Department, Carolina Medical Center, Pory 78, 02-757 Warsaw, Poland; 5 Department of Technical Computer Science, Faculty of Mathematics and Technical Computer Science, Lublin University of Technology, Nadbystrzycka 38, 20-618 Lublin, Poland

**Keywords:** cartilage, EEMD, knee, DFA, acoustic signals, CNN, machine learning, osteoarthritis, biomechanics

## Abstract

This study focuses on the diagnostic analysis of cartilage damage in the knee joint based on acoustic signals generated by the joint. The research utilizes a combination of advanced signal processing techniques, specifically empirical mode decomposition (EEMD) and detrended fluctuation analysis (DFA), alongside convolutional neural networks (CNNs) for classification and detection tasks. Acoustic signals, often reflecting the mechanical behavior of the joint during movement, serve as a non-invasive diagnostic tool for assessing the cartilage condition. EEMD is applied to decompose the signals into intrinsic mode functions (IMFs), which are then analyzed using DFA to quantify the scaling properties and detect irregularities indicative of cartilage damage. The separation of individual frequency components allows for multi-scale analysis of the signals, with each of the functions resulting from the analysis reflecting local variations in the amplitude and frequency over time and allowing for effective removal of noise present in the signal. The CNN model is trained on features extracted from these signals to accurately classify different stages of cartilage degeneration. The proposed method demonstrates the potential for early detection of knee joint pathology, providing a valuable tool for preventive healthcare and reducing the need for invasive diagnostic procedures. The results suggest that the combination of EEMD-DFA for feature extraction and CNN for classification offers a promising approach for the non-invasive assessment of cartilage damage.

## 1. Introduction

According to the World Health Organization (WHO), 528 million people worldwide suffer from degenerative joint diseases. Approximately 73% of those with osteoarthritis are individuals over the age of 55. With the aging of the global population, this number is expected to gradually increase [1]. All the synovial joints have a layer of articular cartilage, which enables smooth, painless and almost frictionless joint movement, properties that are highly affected during OA [2,3,4]. Once the cartilage layer is compromised, the ongoing process of progressive damage is set in motion, and due to the limited cartilage regeneration capacity [5], progressive loss of motion, pain and stiffness of the affected joint develop.

With the progression of osteoarthritis (OA), articular cartilage loses its mechanical properties, such as stiffness, strength, and elasticity. The Young’s modulus decreases, and changes in the extracellular matrix lead to a reduced ability to dissipate loads and increased cartilage permeability. The degeneration of the collagen network and loss of proteoglycans impair the cushioning function, while changes in the water content and friction coefficient accelerate joint damage [6,7,8]. These parameters can be monitored using diagnostic techniques such as MRI or vibroarthrography. Regardless of multiple treatment regiments, including nonsurgical modalities such as activity modification, physical therapy, pharmacological treatment and multiple surgical interventions, to date, OA is an incurable disease. At the end stage of OA, due to pain and restriction of motion, patients are qualified for total joint replacement (TJR). This treatment relieves pain; however, it restricts some daily activities, especially sports and in some areas also working abilities. Moreover, the survival rate of the TJR decreases every year after implantation, and a gross number of patients require revision arthroplasty due to, among others, aseptic loosening of the prosthesis. These findings are of great importance, especially for younger population [9].

At the current stage of medicine, there is no definite cure for OA; however, multiple methods have been described to restore the mechanical properties of cartilage and prolong the joint survival time [1]. These methods can be applied only in the early stage of the disease. Therefore, fast and accurate identification of individuals with cartilage lesions is of high importance. In the diagnosis of OA, conventional radiography is still a workhorse on which treatment options are evaluated. The Kellgren–Lawrence scale [10], which was introduced in the 1950s, enables staging of the disease; however, conventional radiography does not show the cartilage itself but reactive changes in the surrounding bones caused by cartilage loss, such as osteophyte formation, subchondral sclerotization or joint space narrowing. Based on that, effective patient selection for joint-sparring procedures cannot be introduced. High cartilage resolution and acceptable diagnostic accuracy can be obtained with the use of magnetic resonance imaging (MRI). However, this imaging modality is susceptible to several disadvantages, such as high costs, long examination protocol, limited availability, and often underestimation of cartilage lesions [11]. A cheaper and quicker alternative is ultrasound (US). However, due to the joint’s complex anatomy and the inability of sound waves to penetrate bone, gross areas of the joint are blind spots for this modality [12]. Given the above, it seems reasonable to create a new diagnostic modality, which will be effective, non-invasive, fast, and cost-efficient and will not require specialized equipment and highly trained staff.

Vibroarthrography (VAG) seems to fulfill these requirements. Sounds and vibrations emitted during joint motion can be collected and interpreted according to various signal processing methods [13]. To date, there is no strict protocol for data acquisition and processing. Introducing an evidence-based protocol for VAG evaluation can in the future become a screening solution for a large-volume population.

Artificial neural networks (ANNs) are one of the fundamental architectures used in the fields of artificial intelligence and machine learning. Over the past few decades, many different types of neural networks have emerged, each designed to solve specific problems. The two main classes of ANNs are traditional ANNs and convolutional neural networks (CNNs). Although both belong to the same family, they differ in terms of the structure, function, and applications. The principle of operation of convolutional neural networks is similar to traditional artificial neural networks. Like them, CNNs consist of layers of neurons that self-optimize with the progress of learning. One of the most significant limitations of traditional neural networks visible in comparison to the capabilities of CNNs is the tendency to solve tasks of very high complexity. Traditional artificial neural networks (ANNs) are the most classic form of networks, whose basic structure is an input layer, one or more hidden layers, and an output layer. Each neuron in one layer is connected to the neurons in the next layer, and these connections are assigned weights that are adjusted during the training process. These networks are particularly useful for problems where the input data are flattened or transformed into fixed-length vectors, such as analyzing array data or recognizing simple patterns. Convolutional neural networks are designed to solve problems related to the analysis of complex data with a spatial structure, especially in the context of processing complex signals. The main difference between CNNs and traditional ANNs is the way the input data are processed. In convolutional neural networks, data are processed by convolutional layers that apply convolution operations to local regions of the input image, rather than globally combining all the input data as is performed in traditional networks. This allows CNNs to efficiently recognize local patterns, such as edges or textures, and hierarchically combine them into more complex features in subsequent layers. The fundamental difference between the two types of networks is therefore the way in which data are represented and processed. Traditional neural networks rely on the full connection of neurons between layers, which leads to a large number of parameters and limited efficiency for complex, structured data. Convolutional neural networks, on the other hand, thanks to the use of convolution and pooling operations, can better capture local dependencies and are much more efficient in tasks related to image processing, object recognition or segmentation. Convolutional neural networks are a specific type of neural network architecture belonging to the set of deep learning techniques, used on a large scale in modern image detection and classification systems, also in real time [14,15,16,17,18,19].

In our previous studies [20,21,22,23], vibroacoustic signals were analyzed using classical neural networks. At that time, the most commonly used approach involved feature extraction in the form of statistical measures and diagnostic indicators, from which parameters with significant levels of importance were identified using tools such as Student’s t-distribution [24,25], analysis of variance (ANOVA) [26], or neighborhood component analysis (NCA). With the development of advanced tools such as deep learning techniques, which streamline this labor-intensive process, the decision was made to utilize convolutional neural networks (CNNs) for classification. These allow for a faster, automated process of selecting characteristic signal features and reduce the likelihood of missing features important for signal classification.

Recently, transformer models, based on the self-attention mechanism, have gained significant popularity. They revolutionized natural language processing, and their application in image processing is becoming increasingly important. Transformers effectively model global dependencies and are flexible regarding the input size, but they require large datasets and substantial computational resources. On the other hand, CNNs efficiently detect local patterns, are less computationally demanding, and perform well with smaller datasets, although their local nature makes it difficult to model long-range dependencies. Transformers can be challenging to interpret, while CNNs struggle with generalization and often require a fixed input size. Hybrid solutions combine the strengths of both architectures, minimizing their limitations [27,28,29,30].

The generalization approach helps reduce the time required for classification, enabling faster results. This improvement facilitates the rapid screening of larger groups. It forms the foundation for developing non-invasive and cost-effective systems for detecting abnormalities in knee joints during routine examinations, thereby reducing the number of undiagnosed cases. By reducing the effort required for joint condition classification, it creates an opportunity to improve joint health in society through quick diagnostics and the possibility of implementing treatment at earlier stages of degeneration.

This article is a continuation of the research on the diagnosis of osteoarthritis based on vibroacoustic signals. The current work presents an expanded group of study participants as well as a changed approach to the processing and filtering of the analyzed signals, as well as the classification process itself. The approach used not only allowed us to simplify the processing (moving away from the extraction of parameters of biological signals) but also allowed us to improve the classification results.

In the conducted research, deep learning algorithms in the form of convolutional neural networks were used to extract and classify signals recorded for patients diagnosed with chondromalacia of the knee joints and people from the control group. The classification itself was preceded by initial frequency filtering of the signals using EEMD-DFA algorithms. The aim of this paper was to test a new method of filtering and processing vibroacoustic signals to obtain an effective method of classifying the condition of knee joints.

## 2. Materials and Methods

### 2.1. Study Participants

This study was conducted in a group of 97 people, 41 men and 56 women. The average age of the volunteers was 41.67 years. The people participating in the study were divided into two groups: the study group (osteoarthritis, OA), which consisted of patients diagnosed with degenerative disease of the knee joints, and the control group (healthy control, HC). All the patients from the study group were qualified for surgical treatment based on their medical history, physical examination and radiological test results after a prior detailed assessment by a specialist in orthopedics and traumatology of the musculoskeletal system.

The physical examination and vibroarthrography (VAG) used in this study were performed one day prior to the planned surgery, minimizing the time between the procedure and the patient assessment. The HC group consisted of healthy volunteers with no prior knee pain or history of knee injury. The source of the collected and later analyzed data was acoustic signals recorded during research conducted in a group of volunteers in the orthopedics department, as well as in the laboratories of the Lublin University of Technology. This study received a positive opinion from the Bioethics Committee of the Medical University of Lublin, consent number KE-0254/261/2019. This study is a continuation of previous research in an expanded group of participants.

### 2.2. Measurement System and Procedure for Recording Vibroacoustic Signals

**Figure 1:**
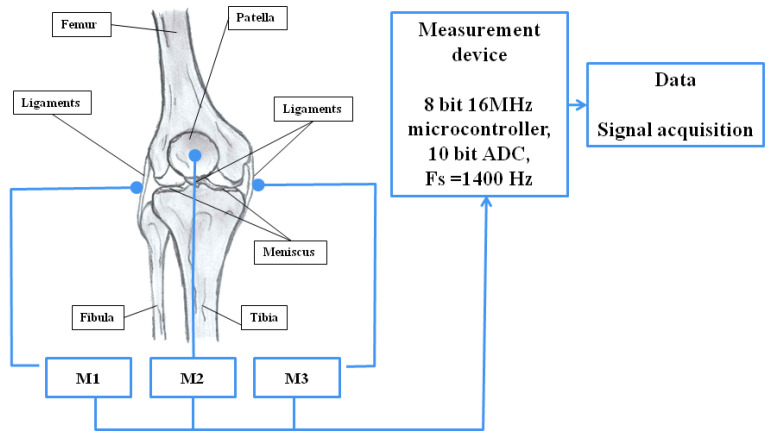
Block diagram of the measurement system.

The measurement system was built on the basis of Arduino Mega2560 R3. Signals were collected using CM01B piezoelectric contact microphones connected to the analog inputs of the Arduino module [31]. The microphones were placed on the patella due to the small amount of soft tissue that could attenuate the signal amplitudes. To ensure patient safety, a galvanic barrier was used on the USB connector, and the device was powered by an 11.1 V lithium-ion battery. The measurement of the angular position (flexion phase) of the limb was performed using the Bourns EMS22A50-D28-LT6 encoder, placed in the rotation axis of the knee joint in the Breg T-Scope Knee Orthosis. The structure of the system is presented in the block diagram shown in Figure 1.

The research protocol included recording signals during a sequence of knee extension and flexion movements from 90° to 0° and back to 90°. The studies were conducted in a closed (CKC) and open (OKC) kinematic chain due to the differences in the biomechanics of movement and different loads on the knee joints in both kinematic chains [32]. The studies conducted in the closed kinematic chain included standing up from a sitting position from knee flexion of 90° to full extension −0°, and then returning to a sitting position with knee flexion to 90°. In the case of the open kinematic chain, the procedure assumed full extension of the limb to 0° from a flexion position of 90°, and then flexion again to an angle of 90°. In both cases, the time for one full cycle of extension from flexion and re-flexion was about 2 s. The signals analyzed in the further part of the article contain 10 cycles described above.

## 3. Preprocessing and Filtering of VAG Signals

This study proposes a hybrid method combining ensemble empirical mode decomposition (EEMD) and detrended fluctuation analysis (DFA) for preprocessing, specifically in the form of filtering input signals [33]. EEMD is designed to decompose complex, nonlinear, and nonstationary signals into a set of intrinsic mode functions (IMFs), which is crucial for analyzing noisy signals, as real-world data often exhibit these characteristics. Artifacts in signal analysis refer to unwanted or spurious components that distort the true signal, leading to inaccuracies or misleading results. These artifacts can arise from various sources, frequently occurring during data collection, and can significantly impact the quality and interpretation of signals. By applying the decomposition technique, the signal is filtered within frequency ranges that are important for further analysis. Subsequently, DFA is used to extract key components relevant to the classification task, which are essential for determining the condition of knee joints. The combination of these methods facilitates effective noise filtering and the extraction of essential diagnostic information from vibroacoustic signals, which is particularly important in biomechanical studies and medicine.

EEMD is a technique used to process nonlinear and nonstationary signals, allowing for the separation of individual frequency bands. It is an improvement over the empirical mode decomposition (EMD) method, as the introduction of white noise to the input signal helps eliminate the undesirable phenomena of amplitude leakage and mode mixing. In addition to limiting aliasing, the inclusion of white noise enhances the stability of the decomposition by reducing the impact of impulse interference [34]. This method is commonly applied in the non-invasive detection of bearing damage, seismic signal analysis, and biomedical data analysis [35,36,37,38]. It has been found that identifying dominant frequency components from vibration signals is often crucial for detecting damage and changes in the dynamic system’s response, both in linear and nonlinear contexts [39,40,41,42].

The EMD algorithm [43] consists of decomposing the signal *x*(*t*) into a number of component functions with specific frequency ranges, called intrinsic mode functions (IMFs). Local maxima and minima are extracted from the input signal. From the obtained values of their amplitudes, two local envelopes are constructed using cubic spline: upper and lower. Their average is determined. The average of the local envelopes is subtracted from the input signal and the component *c*_1_(*t*) is obtained, which is the result of the sieving process. In order to recognize the component *c*(*t*) as a mode (IMF), two conditions are assumed to be met simultaneously:

- The number of extrema and the number of zero crossings must be equal or differ at most by one.
- The mean value of the local envelopes (upper and lower) is equal to zero.

If the conditions are met, the remainder *r*_1_(*t*) is obtained, which serves as an input signal for further decomposition. If the stoppage criterion is not met, the screening process is repeated until the assumed conditions are met. The decomposition is repeated until a monotonic residual signal is obtained. The process can be described as follows:

(1) $$x\left(t\right)=\sum_{i=1}^{n}c_{i}\left(t\right)+r_{n}(t)$$

The improvement of classical EMD to EEMD [44] consists of adding a white noise component of finite amplitude *w_i_*(*t*) to the input signal:

(2) $$x_{i}\left(t\right)=x\left(t\right)+w_{i}\left(t\right),\;$$

where *x*(*t*) is the input signal, *w*_i_(*t*) is a white noise of a length equal to the length of *x*(*t*) and *I* = 1, 2, …, *M*. *M* denotes the number of ensembles.

The decomposition looks similar to that presented for EMD:

(3) $$x_{i}\left(t\right)=\sum_{j-1}^{N}c_{ij}\left(t\right)+r_{i}\left(t\right),$$

where *j* = 1, 2, …, *N*. *N* is the number of IMFs represented by *c*_ij_(*t*) and *r*_i_(*t*) denotes the residual signal of the i-th waveform.

Finally, the ensemble means of the subsequent IMFs are defined:

(4) $$c_{j}\left(t\right)=\frac{1}{M}\sum_{i-1}^{M}c_{ij}\left(t\right).$$

**Figure 2:**
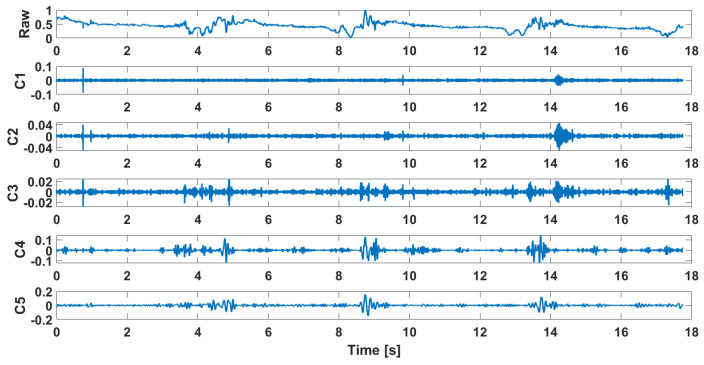
Decomposition results in the time domain, osteoarthritis, M2, closed kinematic chain.

**Figure 3:**
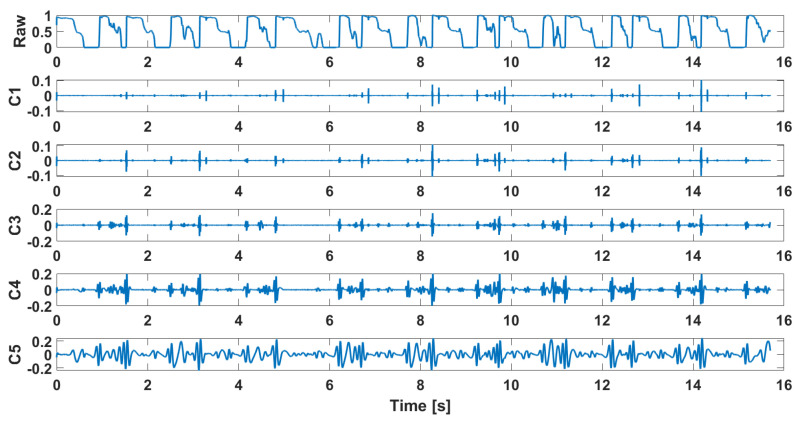
Decomposition results in the time domain, health control, M2, closed kinematic chain.

**Figure 4:**
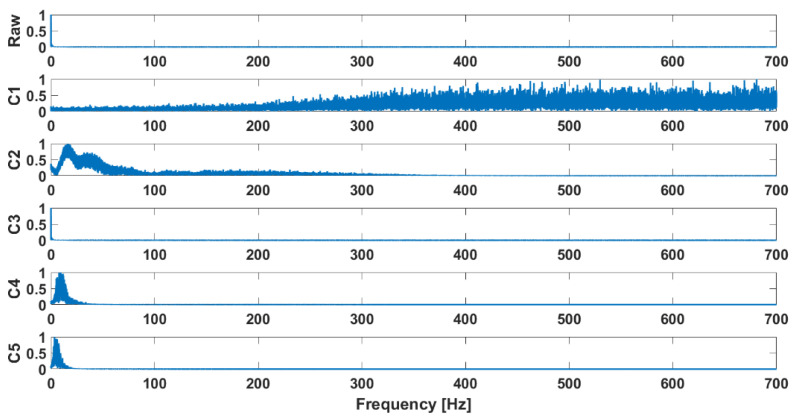
Decomposition results: FFT, osteoarthritis, M2, closed kinematic chain.

**Figure 5:**
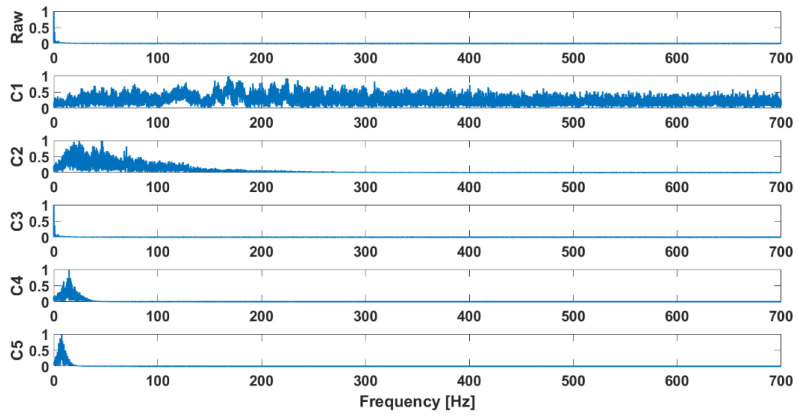
Decomposition results: FFT, health control, M2, closed kinematic chain.

Examples of the waveforms after decomposition obtained for signals recorded in patients diagnosed with chondromalacia of the knee joints are presented in Figure 2, while the decomposition results for the control group are shown in Figure 3. Their FFT is shown in Figure 4 and Figure 5, respectively.

The next step in the analysis of the recorded signals was the implementation of the detrended fluctuation analysis (DFA) part. There are numerous references in the literature regarding the use of this technique in biomedical signals [45,46,47,48,49] due to the rhythmic and repetitive nature of signals originating from the human body. The DFA algorithm is used to detect the occurrence of long-range correlations (LRCs) in the signal. One of the goals of using this technique is also the ability to filter out the useful part of the signal from artifacts [50,51].

Using the DFA algorithm, the fractal scaling factor α is determined, which allows for the description of fluctuations occurring in the signals. The IMF function curves, hereinafter referred to as *q*(*t*), are given separately as the input signal. The DFA algorithm is initiated by determining the average:

(5) $$\bar{q}=\frac{1}{N}\sum_{l=1}^{N}q_{l}(t)$$

Then, the cumulative sum is determined:

(6) $$y\left(m\right)=\sum_{k=1}^{m}\left[q_{k}\left(t\right)-\bar{q}\right]$$

The integrated time series *y*(*m*) is divided into windows of length *n*. In each window, the integrated time series is fitted with a local trend in the form of a polynomial function *y*_f_(*m*). For the present analysis, the value of the polynomial degree is assumed to be one. The detrended fluctuation function *Y*(*m*) is obtained by subtracting the local trend *y*_f_(*m*) from *y*(*m*):

(7) $$Y\left(m\right)=y\left(m\right)-y_{f}(m)$$

The root mean square (rms) fluctuation *F*(*n*) is then calculated for a window of length n:

(8) $$\;F\left(n\right)=\sqrt{\frac{1}{N}\sum_{m=1}^{N}{\left[Y(m)\right]}^{2}}$$

The calculations are repeated for windows of length *n* (lengths change due to different scales), so that the relationship between *F*(*n*) and length *n* is ensured. The existence of a power relation between *F*(*n*) and the selected window size indicates the presence of a scaling relationship: *F*(*n*)~*n^α^*. Using the correlation coefficient, it is possible to obtain certain knowledge related to the nature of the signals and the occurrence of specific properties. In the case where 0.5 < α < 1, we can speak of the occurrence of long-range correlation (LRC), while if the coefficient takes the values 0 < α < 0.5, the signal shows the properties of anticorrelation. If the coefficient takes the value 0.5, the properties of the signal are characteristic of white noise. The presence of α coefficient results greater than 1 indicates that the process is nonstationary.

**Figure 6:**
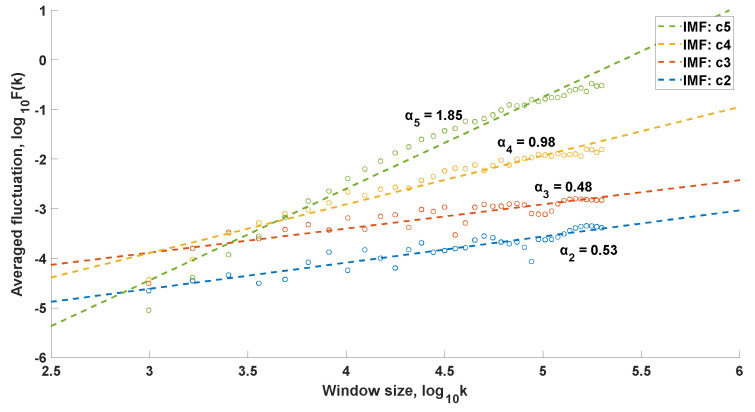
Relationship between the averaged fluctuation and the window length *k*, health control group, open kinematic chain.

**Figure 7:**
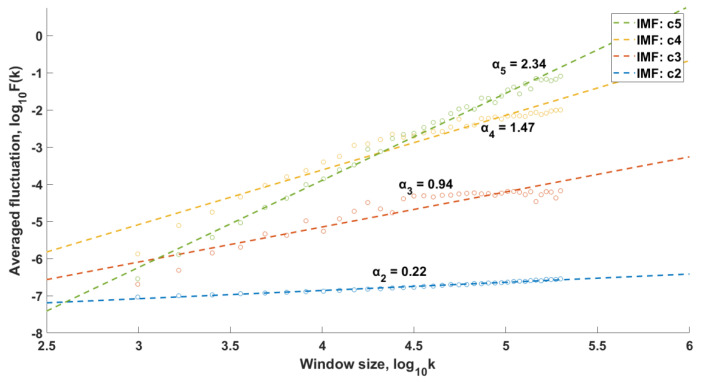
Relationship between the averaged fluctuation and the window length *k*, osteoarthritis group, open kinematic chain.

In the case of the conducted research, the signals resulting from the EEMD procedure (IMF functions) constituted the input data for the DFA procedure. The DFA was performed with window lengths ranging from 50 to 500, with an increment of 10. Figure 6 presents the exemplary relationships between the averaged fluctuation and the window length k for a person from the control group, while Figure 7 shows the corresponding data for a patient diagnosed with chondromalacia of the knee joints. LRC relationships were observed in different IMF components, depending on the type of kinematic chain and the degree of cartilage degeneration within the OA group. Only IMFs exhibiting LRC features were used to reconstruct the signals, which were then passed on for further analysis. These IMFs were extracted using a conditional instruction.

## 4. Analysis and Data Preparation for Classification

The aim of this study was to classify the signals recorded from patients diagnosed with osteoarthritis (OA) of the knee joints and from the healthy control (HC) group. In the problem under consideration, a binary classification was undertaken to distinguish between two groups: healthy people and those diagnosed with osteoarthritis of the knee.

Both groups exhibit different limb movement patterns due to the occurrence of joint stiffness and the presence of pain in the OA group. In the existing literature reports [52,53,54], there is an approach to the analysis of the correlation coefficient α alone in detecting degenerative changes in human gait in progressive Parkinson’s disease. Building on the knowledge gained from analyzing VAG signals recorded in the context of degenerative joint disease, this aspect was incorporated into the current research. After calculating the correlation coefficients for both groups in the closed and open kinematic chains, the median values of the correlation coefficient for each IMF were determined. The difference in the median values of the correlation coefficient between the OA and HC groups was then compared. This comparison enabled the identification of frequency ranges where differences in the long-range correlation (LRC) occur. However, classifying the knee joint condition based solely on differences in the coefficient values was not feasible, as the median differences did not exceed 5% (Table 1).

The obtained values highlight the complexity of classifying vibroarthrographic signals. Contrary to the seemingly simple task of assigning the state of the examined subject based solely on the correlation coefficient, factors such as age, gender, weight, and the individual degree of joint damage complicate the differentiation of the groups. The approach used in the publications referenced above does not work in this case. To achieve more precise and efficient results, it becomes necessary to apply more advanced methods, such as neural networks. The frequency-filtered signal, using EEMD and including components characterized by positive correlations after DFA analysis, was then submitted for further examination. The signal was reconstructed from the extracted components showing significant correlation properties. This process was carried out using a conditional instruction, without rigidly assigning specific IMFs for further analysis.

## 5. Convolutional Neural Networks and Classification

**Figure 8:**
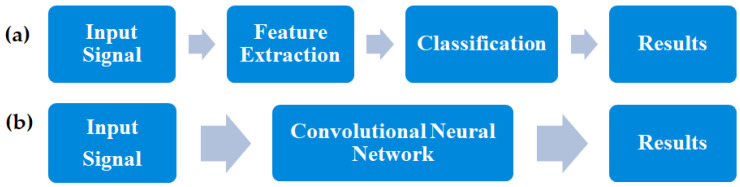
Signal processing (**a**) without CNN and (**b**) with CNN.

Convolutional neural networks are widely used deep learning networks. They are mainly used for image processing, but in recent years, there has been a growing interest in them in the fields of medical signal processing [55,56,57,58,59,60]. Convolutional neural networks (CNNs), also referred to as ConvNets, are a modern research technique that reduces the time-consuming process of manually extracting the characteristic properties of signals. They can be used for both classification and feature extraction, thereby saving time compared to traditional neural networks, which require manual feature extraction (Figure 8).

Classical neural networks are typically composed of three layers: input, hidden, and output layers. The structure of an artificial neural network consists of nodes and connections between them, modeled after the network structure of the human brain. The operation of this structure follows a similar principle. Neurons integrate the input signals and pass them through subsequent connections. The output of each neuron depends on the weighted sum of the signals from previous neurons, which introduces the risk of translation and shift distortions, ultimately affecting the classification accuracy. In the research discussed in this article, a convolutional neural network resistant to translation and shift errors was used.

As with traditional neural networks, the outputs of CNNs depend on the weights and deviations of the previous layers. The most common CNN architecture consists of three layers: convolutional layer (CL), pooling layer (PL) and fully connected layer (FCL). Convolutional layers are the basis for building CNNs. They are composed of kernels that move along the input signal. A kernel is a matrix that interacts with a given input signal. Kernels, sometimes called filters, extract features that distinguish the datasets given at the input. The values of these filters are selected and optimized during network training in order to minimize the network error when solving a given problem. One of the input parameters of this layer is the kernel size. It determines whether the feature extraction will focus on local details with a large amount of information or on more global features. Another input parameter is the padding, which defines the method for selecting samples for analysis and allows adjustment of the network’s input and output size. The stride parameter determines the step by which the sample is shifted in the signal. The result of the convolution process is a specific feature map.

The next layers in the CNN network are the pooling layers. These layers are essential for reducing the dimensions of the data by combining the features from the feature maps to produce more accurate maps. Pooling also counteracts the problems associated with overfitting the model. The final element of a CNN is the fully connected layer. It provides a connection to all the activations in the previous layer.

In the conducted research, to ensure the preservation of the nonlinear nature of the function, the activation functions Rectified Linear Activation (ReLU) and Softmax were used to ensure that the results sum up to the value of 1. The values of the remaining parameters were selected based on the obtained results of the VAG signals so that they corresponded to the nature of the signals tested. The conducted analyses (Table 1) showed that time–frequency dependencies appear in different IMF components, depending on the type of kinematic chain and the individual degree of cartilage damage in the OA group. Filtration allowed us to identify the most important frequency bands for each examined patient, reducing the impact of interference on the classification.

**Figure 9:**
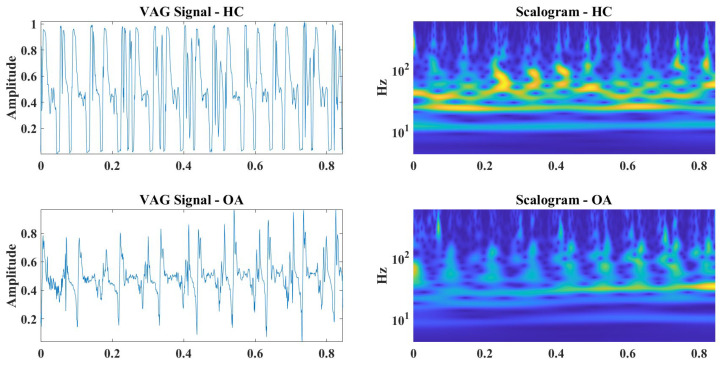
Input data: VAG reconstructed signals and their scalograms, HC and OA, respectively, CKC, M2.

**Figure 10:**
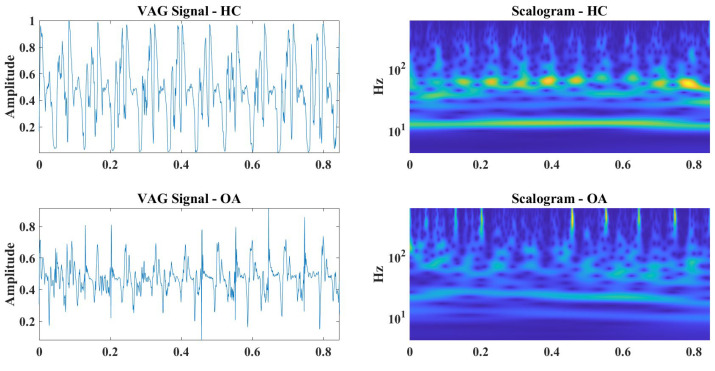
Input data: VAG reconstructed signals and their scalograms, HC and OA, respectively, OKC, M2.

In order to preserve and also take into account the information contained in the frequency domain, a classification method based on the continuous wavelet transform (CWT) and convolutional neural network (CNN) was performed [61]. The decision to use CWT instead of STFT was based on the fact that wavelets are inherently better at handling nonstationary signals and are more effective in analyzing time-varying signals such as speech, music, and biological signals, like the VAG signals studied here. The previously filtered and reconstructed signals were then used to generate the CWT scalogram determinations (Figure 9 and Figure 10), which were treated as input data.

**Figure 11:**
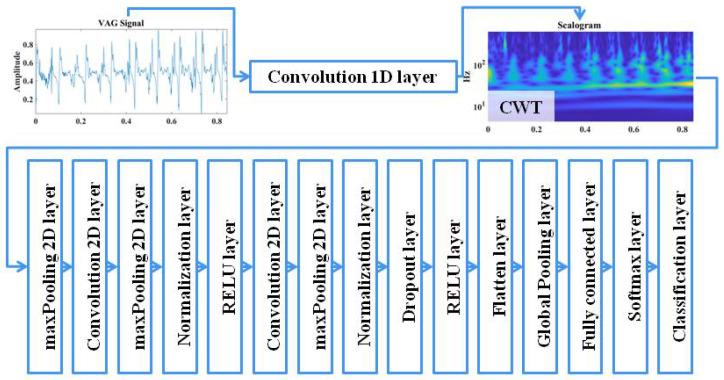
Architecture of the used neural network.

The full network architecture is shown in Figure 11. The regularization parameter value was set to 0.74, the learning rate to 0.001 and the momentum to 0.26. The filter size was set to 5, with 10 filters used. The step value was 2. The best performance within the set time was achieved by specifying the maximum number of epochs as 200 and iterations as 5, which represented a compromise between accuracy and computation time. The input data were divided into a set of training data (70%), validation data (20%) and test data (10%). The calculations were performed using convolutional neural network algorithms in MATLAB R2024b software, based on the CWT-CNN algorithm [61].

Previously conducted experiments on the tasks of the classification of VAG signals using neural networks showed high efficiency of about 90% [20,21,22,23]. The use of convolutional neural networks also gave satisfactory results, as presented in Table 2. The application of EEMD-DFA in the preprocessing and filtering of vibroacoustic signals is justified by their inherent characteristics—these signals often exhibit high nonlinearity and nonstationarity. EEMD facilitated the decomposition of the signal into intrinsic mode functions (IMFs), representing the local frequency components of the signal, adapted to its nonstationary nature. This approach effectively eliminated artifacts associated with traditional methods based on linear analysis. Conversely, DFA enabled the assessment of long-term correlations in the signals, which is critical for detecting subtle changes linked to physiological or pathological processes. The results presented in Table 2 clearly demonstrate that the combination of these methods allows for effective noise filtering and the extraction of significant diagnostic information from vibroacoustic signals, which is of particular importance in biomechanical research and medicine. Furthermore, the classification accuracy obtained for signals processed using the EEMD-DFA procedure was over 30% higher compared to unprocessed signals.

**Figure 12:**
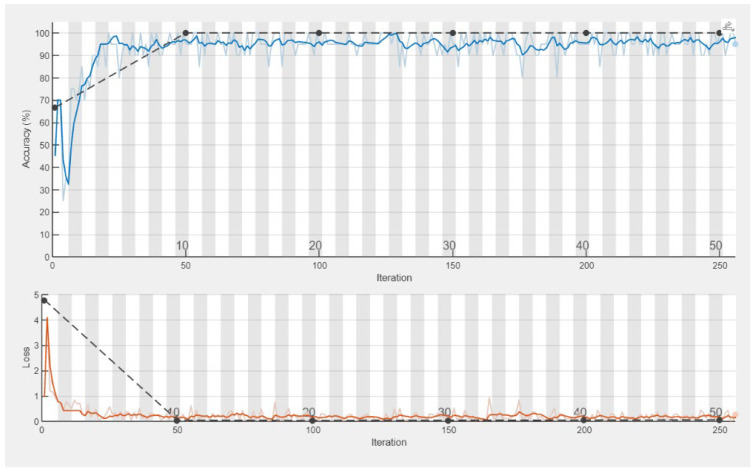
The percentage accuracy of training a convolutional network during the calculation process, EEMD-DFA, M2, CKC. The dashed black line indicates the validation accuracy.

**Figure 13:**
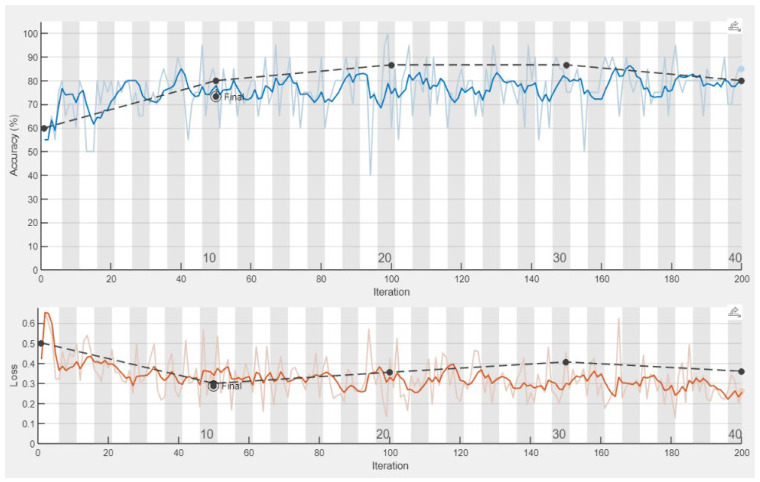
The percentage accuracy of training a convolutional network during the calculation process, raw data, M2, CKC. The dashed black line indicates the validation accuracy.

Figure 12 and Figure 13 show the learning process flow for the data after filtering the frequency components exhibiting positive correlations and for the raw data that were not preprocessed.

## 6. Discussion

Osteoarthritis is one of the most common causes of disability around the globe. It is estimated that due to obesity and aging of the population, more and more individuals will be affected by this disease. To date, there is no definite cure for OA. The available treatment options can prolong the joint survival time and delay need for total joint replacement. In order to achieve this goal, fast and accurate diagnosis of early-stage OA has to be performed. The imaging modalities that are at the disposal of orthopedic surgeons include US, conventional radiography and MRI. Each of these methods has its advantages and drawbacks. However, none of them can be adapted as a large-scale screening modality for OA detection. Vibroarthography seems to fulfill the requirements for an ideal screening tool. It is fast, easy to perform, cheep, can be widely available and no experienced staff are required to perform the examination. Even though VAG is present in heavy industry, it is not ready to be introduced in medicine. The main reason for this is the lack of examination, signal acquisition and processing protocols. Each research group proceeds with different approach toward those issues [62,63,64,65,66].

The processing of vibroacoustic signals recorded within the knee joints encounters a number of difficulties, with the main one being the nonlinear nature of these signals. Knee joints, due to their complex anatomical structure and the presence of various structures, such as cartilage, ligaments and synovial fluid, generate signals of a complex nature. The structure of the vibroacoustic signal is directly dependent on the dynamic interactions between these elements, which causes difficulties in its analysis and modeling. It is emphasized in the literature that nonlinear mechanical phenomena, such as friction, deformation or changes in the viscosity of synovial fluid, can significantly affect the structure of signals, which makes their unambiguous interpretation difficult and requires the use of advanced signal analysis methods, such as wavelet transformations or artificial intelligence algorithms.

A significant difficulty in analyzing vibroacoustic signals is their nonstationarity. These signals change their properties over time, which is the result of dynamic changes occurring in the moving loaded knee joint, such as the changing pressure force during movement or changing lubrication conditions. Due to the nonstationarity of these signals, standard signal analysis methods that assume stationarity become insufficient. Research emphasizes the need to use time–frequency analysis methods that allow for capturing dynamic changes in vibroacoustic signals. Examples of such methods include wavelet transforms or the short-time Fourier transform (STFT), which offer the possibility of more precise modeling of nonstationarity.

The nature of the research object itself remains a challenge—the knee joint is characterized by individual parameters for each patient, which may influence the diversity of vibroacoustic signals. Factors such as age, body weight, degree of cartilage wear, the presence of diseases such as osteoarthritis, and previous injuries can modify the signals significantly. The diversity of these parameters leads to problems with generalizing the results and creating universal diagnostic models. The literature emphasizes that appropriate signal processing techniques must take these variables into account, and the use of learning algorithms such as neural networks can help distinguish specific patterns characteristic of different pathologies.

In this paper, we have tried to utilize EEMD-DFA-CNN as a classification tool for cartilage lesions. Our study showed that over 97% accuracy can be obtained using this approach. Recent advancements in the analysis of vibroacoustic signals have significantly enhanced the diagnostic assessment of cartilage damage. Various studies have explored different combinations of feature extraction methods and machine learning models to improve the classification accuracy. This summary provides a comparative analysis of these methods and their effectiveness, as reported in the literature. However, it is worth mentioning that at this stage, our classification system is only applicable in two-grade classification, namely healthy/injured cartilage. Other research groups also performed two-grade classification of cartilage damage. A summary of the diagnostic accuracy achieved by the various testing teams is detailed in Table 3.

Early research primarily focused on statistical parameters derived from time-domain features. Krishnan et al. [68] utilized statistical parameters in both the time and frequency domains with a logistic regression classifier, achieving an accuracy of 0.77. Rangayyan et al. [69] incorporated clinical features alongside time-domain statistics, improving the accuracy to 0.85 using logistic regression. These studies established a foundation but highlighted the limitations of simpler models and feature sets.

The integration of neural network classifiers marked a significant improvement in the diagnostic accuracy. Rangayyan and Wu [70] employed time-domain statistical parameters with a radial-basis function neural network, reaching an accuracy of 0.91. Similarly, Kim et al. [74] achieved an accuracy of 0.91 using frequency-domain statistics with a back-propagation neural network. These results underscore the capability of neural networks to capture complex patterns in vibroacoustic signals that traditional models might overlook.

Support vector machines (SVMs) and random forest classifiers also demonstrated notable performance. Sharma and Acharya [75] and Yang et al. [76] both used frequency-domain statistical parameters with least square SVMs, achieving accuracies of 0.89 and 0.88, respectively. Shidore et al. [80] employed time- and frequency-domain statistics with a random forest classifier, obtaining an accuracy of 0.89. These machine learning models are effective in handling high-dimensional data and offer robustness against overfitting.

The highest reported accuracy comes from Wang et al. [81], who achieved an impressive 0.98 by combining kernel-radius-based features with statistical features and utilizing a back-propagation neural network. This suggests that innovative feature engineering, when paired with advanced neural network architectures, can significantly enhance diagnostic performance.

Recent studies have continued this trend of improvement. Machrowska et al. used recurrence indicators with multilayer perceptron and radial basis function networks, reaching an accuracy of 0.91. Kręcisz and Bączkowicz [78] achieved an accuracy of 0.90 by integrating time-domain features with nonlinear statistics in a logistic regression model enhanced by automatic attribute selection.

Two methods for analyzing AE signals were compared: manually extracted features (accuracy of 90 ± 7.16%) and deep wavelet scattering (DWS), which achieved higher accuracy at 97 ± 3.77%. DWS proved to be more effective but more computationally demanding. The analysis covered five types of joint pathologies, with the best results obtained using the LDA and SVM models [88]. In the study [89], it was demonstrated that the synchronization of biomechanical and acoustic signals achieved 93% accuracy in classifying different lubrication modes.

In the article [90], the analysis included feature extraction in the time and frequency domains, as well as the application of machine learning algorithms for classifying the joint health status. The highest accuracy was achieved using SVM, which exceeded 90% in distinguishing between healthy and diseased joints. Using supervised machine learning, including logistic regression, k-nearest neighbor (KNN), and back-propagation (BP) neural networks, the research achieved high classification accuracy, with BP neural networks performing best at 98% [91]. Principal component analysis (PCA) reduced the data dimensionality while retaining 95% of the variance. AE signals effectively distinguished between adhesive and abrasive wear, showing potential for developing diagnostic tools for joint health monitoring and implant assessment [91]. The article [92] presents the use of acoustic emission (AE) analysis with an unsupervised graph algorithm as a tool for assessing knee health. A key finding was the introduction of the Graph Community Factor (GCF), which demonstrated differences between healthy participants (18.5 ± 3.5), individuals with acute knee injury (24.8 ± 4.4), and the post-rehabilitation state (16.5 ± 4.7) [92].

The authors of the study [93] also conducted tests using two models: a feature-based model and a convolutional neural network (CNN). Both models were trained on data from healthy participants and patients with RA and then tested on a dataset including healthy individuals, those with preradiographic OA (Pre-OA), and OA. The feature-based model achieved an AUC of 0.69 for Pre-OA and 0.94 for OA, sensitivity of 0.38 (Pre-OA) and 0.80 (OA), and specificity of 1. The CNN delivered better results, with an AUC of 0.85 for Pre-OA and 0.99 for OA, sensitivity of 0.50 (Pre-OA) and 1 (OA), and specificity of 0.90 [93]. Recent studies utilizing deep neural networks have demonstrated their effectiveness in medical diagnostics based on the classification of vibroarthrography (VAG) signals [86,87,94]. In the study by Jong et al. [86], the authors proposed a method for quantifying the asymmetry of the knee joint load between the medial and lateral compartments in healthy individuals. This method utilized the time–frequency representation of knee acoustic signals (cross-spectrum and wavelet coherence) combined with the CNN and SVM models, achieving a classification accuracy of 83%. Zhang et al. [87] demonstrated that multiscale feature extraction enabled the monitoring of multi-grade osteoarthritis deterioration using VAG signals and confusion-free master–slave (CF-MS) classification, achieving an accuracy of 77%. Additionally, the time–frequency representation of VAG signal properties using spectrograms, along with data augmentation, allowed for excellent classification results with CNN models (AUC = 87% and F1-SCORE = 80%) [94].

In comparison, studies relying solely on time-domain features or simpler classifiers like logistic regression generally reported lower accuracies. For instance, Krishnan et al. [72] and Wu and Krishnan [73] achieved accuracies of 0.84 and 0.80, respectively. This highlights the importance of both sophisticated feature extraction methods and advanced classifiers in improving diagnostic outcomes.

The adopted approach, in comparison to the results presented by other authors, integrates the processing of VAG signals both in the time domain (empirical mode decomposition (EEMD) combined with filtering and reconstruction) and in the time–frequency domain (wavelet analysis) with convolutional neural network (CNN) classification algorithms. The extraction of the dominant frequency components highlights the most informative features of the analyzed vibroacoustic signals, which vary depending on the condition of the knee joint. Particularly important is the filtering and algorithmic determination of the most significant frequency components, where the literature often employs a subjective selection approach (e.g., choosing the first few components).

Vibroacoustic analysis of knee joints is an innovative, non-invasive method of research conducted based on signal analysis. The conducted studies have shown the effectiveness of the hybrid EEMD-DFA-CNN method in the classification of vibroacoustic signals recorded for patients with chondromalacia of the knee joints and the control group. Despite obtaining good results, it requires further research in a larger group, taking into account both a larger number of cases and different degrees of cartilage tissue damage.

As an additional aspect of this research, the lack of significant correlation between the condition of the knee joints and the obtained correlation coefficient α was noticed. The differences in the median values of the obtained coefficient values between the patients and the control group did not exceed 5%. However, it was valuable to determine the most important frequency filtering ranges. It was shown that the input signal without filtration does not show a greater correlation than the signal from the individual bands. This will allow for more effective selection of analysis ranges in the future.

In conclusion, the comparative analysis indicates a clear progression toward higher accuracies in cartilage damage diagnostics using vibroacoustic signal classification. The most significant improvements are associated with the use of advanced neural networks and the development of specialized features that capture the underlying complexities of the signals. Future research should continue to explore innovative feature extraction techniques and leverage cutting-edge machine learning models to further enhance the diagnostic potential of vibroacoustic analysis in medical applications.

The main limitations of the proposed method observed during the conducted studies undoubtedly include the sample size and the diversity of the groups.

To fully evaluate the analyzed issue and leverage the potential of the proposed method, further research should be conducted on a significantly larger number of cases, taking into account the complete four-grade scale according to the ICRS classification and the precise anatomical locations of degenerative changes. This would allow for obtaining detailed results regarding the degree of damage and the zones of articular cartilage damage, as well as for developing an advisory system enabling comprehensive diagnostics.

Additionally, the analyses should also include damage to other knee joint structures, such as the menisci or ligaments, as these structures are often associated with the occurrence of osteoarthritis.

## 7. Conclusions

This study focuses on the diagnostic assessment of cartilage damage in the knee joint by analyzing acoustic signals generated during joint movements. It uses advanced algorithms, in particular ensemble empirical mode decomposition–detrended fluctuation analysis (EEMD-DFA) and convolutional neural networks (CNNs), to process and interpret these signals. The main goal was to identify and classify cartilage degradation based on the unique patterns found in acoustic emissions, which are influenced by the mechanical properties of the cartilage and joint surfaces.

The EEMD-DFA algorithm is used to decompose the acoustic signal into IMF modes and perform preliminary filtration of the data containing characteristic features, while the CNN model is trained to recognize patterns indicative of damage. This combined approach increases the sensitivity and accuracy of detecting early-stage cartilage damage, offering a non-invasive method for monitoring joint health in real time. The results suggest that this methodology may be a promising tool for diagnosing knee joint diseases, particularly osteoarthritis, by analyzing sound emissions during normal joint movement. Comparative studies are planned using transfer learning by leveraging pre-trained models on large datasets, followed by feature extraction with their help, as well as fine-tuning.

A novelty of the conducted research was the reduction of computational effort by using filtered signals as input in CNNs, bypassing the step of parameter estimation and determining their relevance in the analysis. The applied solution allows for a reduction in computational complexity, enabling faster results while maintaining high computational accuracy. A valuable insight for both existing and future computational studies is the conclusion that classifying VAG signals based solely on the correlation coefficient is not feasible. The research demonstrated that the α coefficient provides valuable information useful for frequency filtering applications, but it is insufficient on its own to perform a simplified classification of joint condition based only on its value. In conclusion, this study presents a novel, data-driven approach for the early detection of cartilage lesions, using the synergy between signal processing techniques and machine learning to provide a reliable, efficient diagnostic tool.

## Figures and Tables

**Table 1:** Percentage differences in the median values of the correlation coefficient between the group of patients diagnosed with chondromalacia of the joints and the control group. IN: input signal, M1, M2, M3: sensor locations.

Signal	Closed Kinematic Chain			Opened Kinematic Chain		
	M1	M2	M3	M1	M2	M3
IN	0.121	0.268	0.112	0.088	0.046	0.026
IMF 1	1.113	1.297	0.493	0.231	0.786	0.201
IMF 2	4.977	1.811	2.202	1.612	0.085	0.030
IMF 3	0.103	2.041	2.989	4.067	4.207	2.881
IMF 4	0.052	4.438	0.204	0.452	4.168	0.769
IMF 5	0.207	1.820	0.157	1.358	1.106	0.093
IMF 6	0.179	0.543	0.233	0.266	0.781	0.028
IMF 7	0.051	0.101	0.048	0.127	0.081	0.024
IMF 8	0.011	0.080	0.023	0.045	0.004	0.010
IMF 9	0.028	0.040	0.021	0.009	0.021	0.026
IMF 10	0.030	0.041	0.025	0.021	0.036	0.005
IMF 11	0.012	0.004	0.012	0.016	0.024	0.016
IMF 12	0.010	0.003	0.005	0.012	0.010	0.011

**Table 2:** Accuracy of CNN learning. M1, M2, M3: microphones localizations.

	Accuracy [%]	Closed Kinematic Chain			Opened Kinematic Chain		
		M1	M2	M3	M1	M2	M3
Raw data	Learning	74.6	77.3	73.7	73.2	75.8	65.7
	Validation	71.3	73.3	69.7	72.1	67.3	63.4
	Testing	70.6	71.6	68.5	70.2	63.7	58.3
EEMD-DFA filtration	Learning	99.1	100	98.7	99.4	100	98.5
	Validation	98.7	99.3	98.5	99.2	98.6	98.3
	Testing	98.6	98.9	98.3	99.1	98.4	97.8

**Table 3:** Comparison of the diagnostic accuracy obtained by research groups.

Authors and Sources	Features	Model	ACC
Rangayyan and Wu [67]	Statistical parameters in time domain	Neural network classifier based on radial basis functions	0.82
Krishnan et al. [68]	Statistical parameters in time and frequency domains	Logistic regression classifier	0.77
Rangayyan et al. [69]	Statistical parameters in time domain and a clinical features	Logistic regression classifier	0.85
Rangayyan and Wu [70]	Statistical parameters in time domain	Neural network classifier based on radial-basis functions	0.91
Rangayyan and Wu [71]	Statistical parameters in frequency domain	Neural network classifier based on radial-basis functions	0.82
Krishnan et al. [72]	Autoregression coefficients	Logistic regression classifier	0.84
Wu and Krishnan [73]	Statistical parameters in frequency domain	Recurrent neural network	0.80
Kim et al. [74]	Statistical parameters in frequency domain	Back-propagation neural network	0.91
Sharma and Acharya [75]	Statistical parameters in frequency domain	Least square support vector machine	0.89
Yang et al. [76]	Statistical parameters in frequency domain	Least square support vector machine	0.88
Wu et al. [77]	Statistical parameters in time and frequency domains	Support vector machine	0.83
Kręcisz and Bączkowicz [78]	Statistical parameters in time, nonlinear statistics	Logistic regression classifier with automatic attribute selection	0.90
Athavale and Krishnan [79]	Statistical parameters in time and frequency domains	Support vector machine	0.84
Shidore et al. [80]	Statistical parameters in time and frequency domains	Random forest classifier	0.89
Wang et al. [81]	Kernel-radius-based feature and statistic-based feature	Back-propagation neural network	0.98
Mascarenhas et al. [82]	Statistical parameters in time and frequency domains	Random forest classifier	0.86
Moreira et al. [83]	Statistical parameters in time and frequency domains	K-nearest neighbors classifier	0.89
Machrowska et al. [84]	Recurrence indicators	Multilayer perceptron, radial basis function	0.91
Karpiński [85]	Statistical parameters in time and frequency domains	Multilayer perceptron, radial basis function	0.90
Jeong et al. [86]	Time and frequency domains	Convolution neural network, support vector machine	0.83
Zhang et al. [87]	Frequency domain	Convolution neural network, confusion-free master–slave	0.77

## Data Availability

The data presented in this study are available from the corresponding author upon request.
